# MiLaSol: modeling protein solubility by mixing up multiple protein language models

**DOI:** 10.1093/bioadv/vbag233

**Published:** 2026-08-13

**Authors:** Weiwei Lou, Mert Erden, Lenore Cowen

**Affiliations:** Department of Computer Science, Tufts University, Medford, MA 02155, United States; Department of Computer Science, Tufts University, Medford, MA 02155, United States; Department of Computer Science, Tufts University, Medford, MA 02155, United States

## Abstract

**Motivation:**

Protein solubility is a critical property that significantly impacts therapeutic efficacy and protein reengineering applications. Recent advances in machine learning and deep learning techniques provide unprecedented opportunities to develop predictive models for solubility, enabling more efficient protein design and optimization. This work is motivated by the potential of leveraging deep learning to address the solubility prediction challenge and to accelerate protein engineering workflows.

**Results:**

Leveraging and combining multiple protein language model representations, our MiLaSol model attains 81% accuracy, outperforming prior methods, with the highest Matthews Correlation Coefficient (MCC) score of 0.63 demonstrating balanced performance across both soluble and insoluble proteins. Through simulated annealing optimization coupled with the Raygun model, we also present a computational method to reengineer insoluble protein variants into soluble forms, with predictions confirmed by multiple independent solubility prediction methods. Our results demonstrate the effectiveness of combining machine learning-based solubility prediction with generative optimization for protein engineering.

**Availability and implementation:**

MiLaSol is available at https://github.com/weiweiloutufts/milasol

An archived version of the code at the time of submission can be found at https://doi.org/10.5281/zenodo.21495202

## 1 Introduction

Protein solubility plays a vital role in biotechnology, biochemistry, and medicine, particularly in the expression and purification of therapeutic proteins, affecting their stability, functionality, and bioavailability (Qing *et al.* 2022). For essential cellular processes such as DNA replication, RNA transcription, and ribosomal protein translation to occur efficiently, proteins must be water-soluble in the aqueous cytosol. In therapeutics, solubility is critical in determining the suitability of candidate antibodies and peptides.

Protein solubility is influenced by a variety of extrinsic and intrinsic factors. Extrinsic factors include solution pH, temperature, solvent type, ionic strength, metal-ion cofactors, and the presence of surfactants. The intrinsic factors include protein size, molecular hydrophobicity, electrostatic properties, charge distribution, and fraction of exposed residues (Qing *et al.* 2022). Because solubility can be measured with variable extrinsic factors, published measures of intrinsic protein solubility are difficult to calibrate and not fully comparable. Numerous studies have nonetheless sought to predict intrinsic protein solubility directly from amino acid sequence or to understand how sequence variations, such as deletions, insertions, or point mutations, impact solubility. However, even after controlling for extrinsic factors, learning a reliable score of intrinsic protein solubility directly from the amino acid sequence has proved quite difficult, since the intrinsic solubility is sometimes seemingly dependent on global features of the amino acid sequence, and sometimes seemingly dependent on more localized regions of the protein sequence or structure. For example, although both hemoglobin and G protein-coupled receptors (GPCRs) are rich in α-helical content, hemoglobin is highly water-soluble, while GPCRs are water-insoluble (Qing *et al.* 2022).

Early computational methods to predict protein solubility directly from amino acid sequence relied on hand-crafted features that come from known physicochemical properties (Agostini *et al.* 2012, Smialowski *et al.* 2012, Sormanni *et al.* 2015, Bhandari *et al.* 2020). Prior deep learning approaches have typically fused together these hand-crafted features that come from known physicochemical properties of solubility with features automatically extracted from large language models (Khurana *et al.* 2018, Rawi *et al.* 2018, Wu and Yu 2021). With the advancement of AlphaFold and its successors and competitors to accurately predict protein structures, some researchers have begun to incorporate structural information also into their work (Tan *et al.* 2024b). However, many existing approaches struggle to integrate structural information effectively, despite its importance in capturing the physicochemical properties that govern solubility.

In this study, we introduce MiLaSol (*Mi*xup *La*nguage *Sol*ubility), a new deep learning model that aims to predict protein solubility. The strength of MiLaSol comes from its incorporation of multiple different PLM-based embeddings: amino acid sequences are utilized in conjunction with differential embeddings derived from the pre-trained ESM2 (Lin *et al.* 2022), Raygun (Devkota *et al.* 2024) and ProtT5 (Elnaggar *et al.* 2021) protein language models (PLMs). These are all input into our proposed model. We find that using and combining signals from multiple different pre-trained language models allows us not to need to include hand-tuned solubility-related features: our model is able to learn what it needs to know without them. We find on standard popular solubility benchmarks that MiLaSol significantly outperforms previous state-of-the-art methods for this problem.

Beyond prediction, we show that MiLaSol can guide the design of more soluble protein variants. Because no benchmark exists that directly measures mutations converting insoluble proteins to soluble ones, we evaluate this capability using SoluProtMutDB (Veleckỳ *et al*. 2022), a curated database of soluble proteins annotated with the experimentally determined solubility effects of point mutations, scored on a five-category scale from strongly solubility-increasing (++) to strongly solubility-decreasing (– –). We treat variants with maximally deleterious (– –) effects as insoluble starting points and use one of MiLaSol’s internal PLM embeddings, Raygun, to computationally re-engineer them toward improved solubility.

## 2 Related work

Various computational methods have been developed for protein solubility prediction over the past three decades. Table 1 summarizes the key characteristics of existing approaches, highlighting the evolution from simple statistical methods to sophisticated machine learning algorithms. Most methods focus on *E. coli* expression systems and utilize sequence-based features, with recent approaches incorporating advanced feature engineering and deep learning techniques.

**Table 1:** Previous protein solubility prediction methods.

Method	Year	Algorithm/approach	Key Features
RPSP (Wilkinson and Harrison 1991)	1991	Statistical analysis	Six compositional parameters
PROSO (Smialowski et al. 2007)	2007	Two-layer model (SVM → Naive Bayes)	Amino acid composition
SOLpro (Magnan et al. 2009)	2009	Two-stage SVM classifier	Physicochemical properties and predicted structural features
CCSOL (Agostini et al. 2012)	2012	Support Vector Machine	Physicochemical properties and amino acid composition
PROSO II (Smialowski et al. 2012)	2012	Two-layer model (Parzen window + logistic regression)	Physicochemical properties and amino acid composition
SCM (Huang et al. 2012)	2012	Scoring card method	Dipeptide composition
CamSol (Sormanni et al. 2015)	2014	Statistical Calculation	physicochemical properties
PaRSnIP (Rawi et al. 2018)	2018	Gradient Boosting Machine	57 engineered features: amino acid composition, predicted structural properties, and physicochemical attributes
DeepSol (Khurana et al. 2018)	2018	Multi-convolution-pooling	One-hot encoded sequence and PaRSnIP engineered features
SWI (Bhandari et al. 2020)	2020	Arithmetic mean	Protein sequence properties
EPSOL (Wu and Yu 2021)	2021	Multi-convolution-pooling	Raw sequence, secondary structure, and PaRSnIP engineered features
PLM _ Sol (Zhang et al. 2024)	2024	biLSTM-TextCNN	Raw sequence and ProtT5 encoder

In recent years, numerous studies have explored the integration of Convolutional Neural Networks (CNNs) and Long Short-Term Memory (LSTM) networks in the computational biology field. One notable example is DeepSol (Khurana *et al.* 2018) which leverages this combination to predict protein solubility and demonstrates effective performance.

DeepSol employs a large-scale benchmark training dataset derived from experimentally validated protein solubility data compiled by Smialowski *et al.* (2012). This dataset contains 69 420 protein sequences with confirmed solubility outcomes from laboratory experiments conducted primarily using *E. coli* expression systems. The sequences were sourced from the pepcDB database (Berman *et al.* 2009), which tracks experimental results from various protein research centers, and the Protein Data Bank (PDB) where proteins are annotated with their expression conditions. Of these sequences, 28  are labeled as soluble (successfully expressed and remained in solution) while 40  are classified as insoluble (formed aggregates or precipitates during expression). The DeepSol authors applied several data cleaning steps including removing membrane proteins, reducing sequence similarity to 90% maximum identity using CD-HIT (Li and Godzik 2006, Fu *et al.* 2012) clustering, and ensuring no overlap between training and test sequences. For model evaluation, DeepSol uses the test dataset established by Chang *et al.* (2014), which has become the standard evaluation set in the protein solubility prediction field. This test set contains 2001 sequences (1000 soluble and 1001 insoluble) that were compiled from multiple existing solubility databases including PROSO II (Smialowski *et al.* 2012) and PROSO (Smialowski *et al.* 2007). The test sequences were processed to remove redundancy at 30% sequence identity level and represent a balanced evaluation framework. DeepSol’s use of this widely-adopted benchmark allows for direct performance comparison with other established methods such as PaRSnIP (Rawi *et al.* 2018), EPSOL (Wu and Yu 2021) and traditional machine learning approaches, making it possible to assess the relative effectiveness of different solubility prediction strategies using consistent evaluation criteria.

DeepSol achieved 77% accuracy on the authors’ benchmark dataset, outperforming contemporary solubility predictors. More recently (2021), EPSOL (Wu and Yu 2021) used the same dataset and surpassed DeepSol with 79% accuracy, while proposing also benchmarking against an independent test set provided by Chang *et al.* (2014); together, these datasets became a widely adopted gold standard, against which most subsequent methods—including ours—now benchmark performance.

In addition, the Deep_CNN_LSTM_GO framework (Elhaj-Abdou *et al.* 2021) also employs both CNN and LSTM architectures to forecast protein functions. We further note that the use of the PLMs for protein sequence representation, as in MiLaSol, in methods that predict protein solubility is widespread. For instance, Tan *et al.* (2024b) utilize frameworks such as ESM2 (Lin *et al.* 2022), ProtBert (Brandes *et al.* 2022), ProtT5 (Elnaggar *et al.* 2021), or Ankh (Elnaggar *et al.* 2023) to encode amino acid sequences. They also employ roto-translation equivariant graph neural networks to enhance local interactions by modeling the protein backbone. In another study, Tan *et al.* (2024a) generate structural sequence representations using FoldSeek (van Kempen *et al.* 2022) and DSSP (Hekkelman *et al.* 2025), which they then convert into dense vectors for downstream tasks. Their findings indicate that incorporating structural information significantly improved accuracy by 10% compared to using a standard sequence-based PLM. Interestingly, even with this structural information, the prediction accuracy remained lower than that of DeepSol.

## 3 Method

### 3.1 Model architecture

In downstream protein analysis, prior work often enhances protein language model (PLM) representations with manually curated features such as physicochemical descriptors, structural labels, contact maps, or handcrafted sequence statistics. While effective, such approaches rely heavily on domain expertise and introduce additional preprocessing overhead. In contrast, MiLaSol operates exclusively on PLM-derived representations, eliminating the need for handcrafted features while achieving competitive or superior predictive performance.

**Figure 1:**
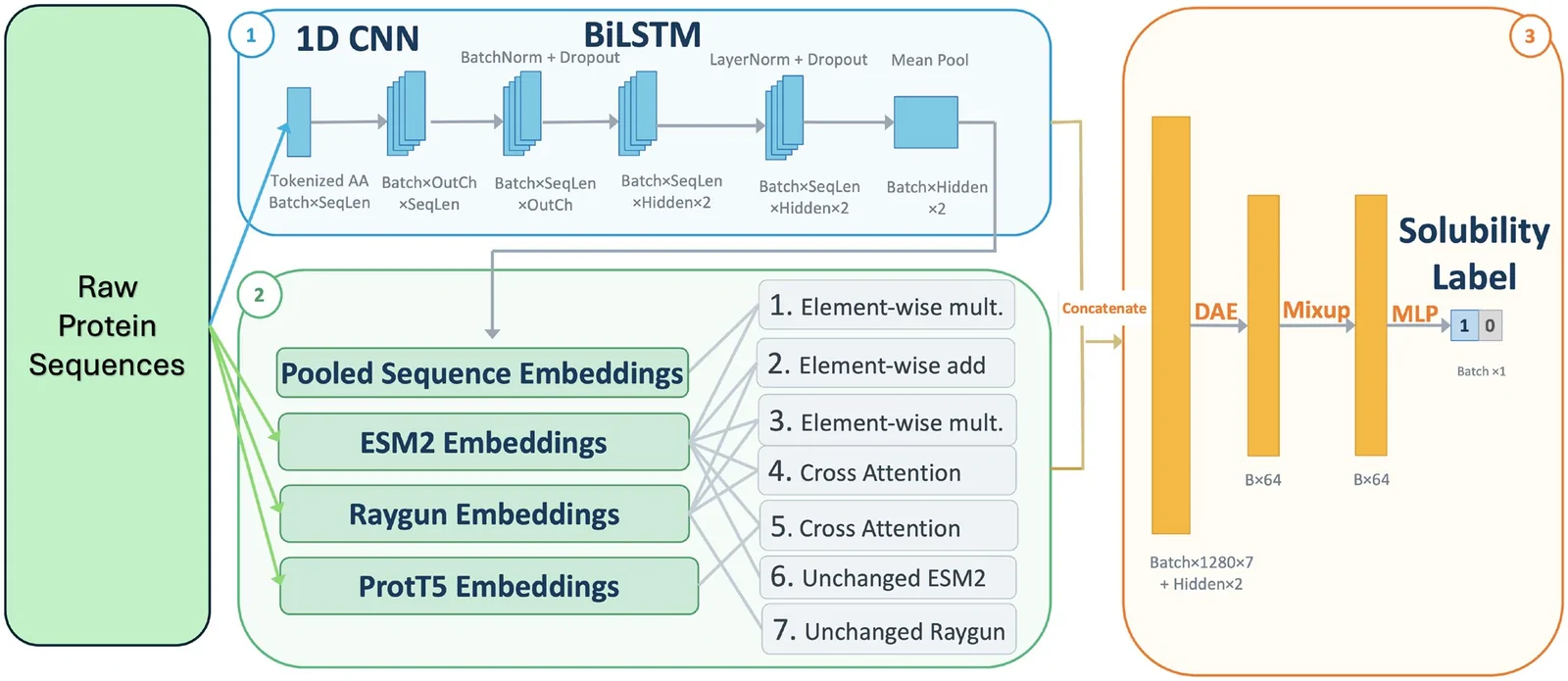
Architecture of MiLaSol. Part 1: Sequence tokens are processed using a 1D CNN followed by a BiLSTM to capture a local and global sequence context; Part 2: Complementary representations are integrated from ESM2, Raygun, and ProtT5 models; Part 3: Features are fused and regularization is applied using a denoising autoencoder (DAE) with mixup-based latent-space augmentation. The resulting latent representations are passed through an MLP for binary classification.

MiLaSol’s architecture consists of three major components: (1) Multi-view sequence encoding, (2) Representation augmentation, and 3) Classification architecture as described in more detail next, and summarized in Fig. 1.

#### 3.1.1. Multi-view sequence context encoding

To effectively capture both local and global sequence dependencies, MiLaSol combines convolutional and recurrent modeling strategies. Inspired by prior successes (Budach and Marsico 2018, Khurana *et al.* 2018, Elhaj-Abdou *et al.* 2021) in protein sequence modeling, we use a convolutional layer that aims to extract localized biochemical and structural motifs from the input embeddings.

The model operates on four complementary input representations:

- Tokenized amino acid sequences
- ESM2 (Lin *et al.* 2022) embeddings
- Raygun (Devkota *et al.* 2024) embeddings
- ProtT5 (Elnaggar *et al.* 2021) embeddings

The three PLM embeddings are pooled, sequence-level representations: they capture global sequence context but do not preserve residue-level positional detail. We retain the tokenized amino-acid sequence as a separate input precisely so that the CNN and BiLSTM can recover this local, residue-level context that the pooled embeddings discard. Removing the tokenized sequence entirely, leaving only the three pooled PLM embeddings, lowers accuracy from 0.81 to 0.70 and MCC from 0.63 to 0.42 (Table S11, available at supplementary material  *Bioinformatics Advances* online), confirming that the pooled embeddings alone are insufficient.

Following Khurana *et al.* (2018) each input representation is projected into a unified latent space via a learnable linear projection. A 1D convolution is then applied to capture local contextual information producing representations $s\in R^{n\times k}$, where *k* denotes the number of convolutional filters and *n* is the sequence length.

To model long-range dependencies, the convolved features are passed through a bidirectional LSTM. The resulting sequence representations are aggregated via scaled dot-product self-attention, followed by mean pooling on the length dimension, yielding a fixed-dimensional embedding in $R^{p}$ that summarizes the global sequence context.

#### 3.1.2. Multi-representation interaction

To improve robustness and generalization, MiLaSol incorporates a structured data augmentation strategy that exploits complementary information across multiple PLM embeddings. Specifically, we utilize three pretrained encoders, ESM2, ProtT5, and Raygun. Through experimentation we find that these representations complement each other, and are sufficient in matching the performance of structure-based and hand-crafted feature models.

We define five interaction mechanisms to integrate information across representations:

1. *Element-wise Multiplication* Interaction: ESM2 embedding ⊙ projected BiLSTM output
2. *Additive Fusion*: ESM2 embedding + Raygun embedding
3. *Multiplicative Fusion*: ESM2 embedding ⊙ Raygun embedding
4. *Cross Attention*: Raygun embeddings attend ESM2 embeddings
5. *Cross Attention*: Raygun embeddings attend ProtT5 embeddings

#### 3.1.3. Classification architecture

All generated embeddings are concatenated to form a unified representation:


(1) $$e_{\mathrm{fusion}}=e_{\mathrm{ESM}2}⊕e_{\mathrm{Raygun}}⊕e_{\mathrm{BiLSTM}}⊕\overset{5}{\underset{i=1}{⊕}}e_{\mathrm{interaction}}^{\left(i\right)}$$

where ⊕ denotes the concatenation operation.

The fused embedding is passed through a two-layer multilayer perceptron (MLP) prediction head with tanh activation and dropout (rate = 0.3). The output of this layer is a logit associated with predicting whether or not the protein is soluble.

### 3.2 Regularization and representation stabilization

To enhance generalization and robustness, we apply a series of complementary regularization strategies that operate at both the representation and optimization levels. These techniques are designed to stabilize training, reduce overfitting, and encourage semantically meaningful latent spaces.

#### 3.2.1 Denoising autoencoder regularization

Inspired by successes in semi-supervised learning (Vincent *et al.* 2008), we introduce an auxiliary denoising task during training to encourage the model to have a smooth latent space.

A denoising autoencoder (DAE) (Vincent *et al.* 2008) is employed on the latent space of the fused representation. Gaussian noise is injected into the embedding,


$$\tilde{e}=e+\epsilon ,\;\epsilon ∼N(0,0.1^{2}I),$$

and the model is trained to reconstruct the original representation via an L2 reconstruction loss:


$$L_{\text{recon}}=\|\hat{e}-e{\|}_{2}^{2}.$$

This encourages the network to be robust to small perturbations around the training representations, since the decoder must map noise-corrupted embeddings back to their clean counterparts. We note that this local robustness is distinct from the global continuity associated with probabilistic latent models such as VAEs, which enforce an organized latent via a prior and are themselves not fully smooth in practice (Li *et al.* 2021).

#### 3.2.2 Latent mixup augmentation

To further regularize the learned representations, we apply mixup (Zhang *et al.* 2017) directly in the latent space. To encourage smooth interpolation in the latent space, we applied a convex combination of each embedding with a randomly sampled counterpart, defined as


$$e_{\mathrm{aug}}=\lambda e+(1-\lambda )e_{\text{index}},\;\lambda \in [0.75,0.95)$$

where λ was drawn uniformly from the specified interval. We find that these augmentations both produce more information dense representations while also generalizing better on solubility prediction.

**Figure 2:**
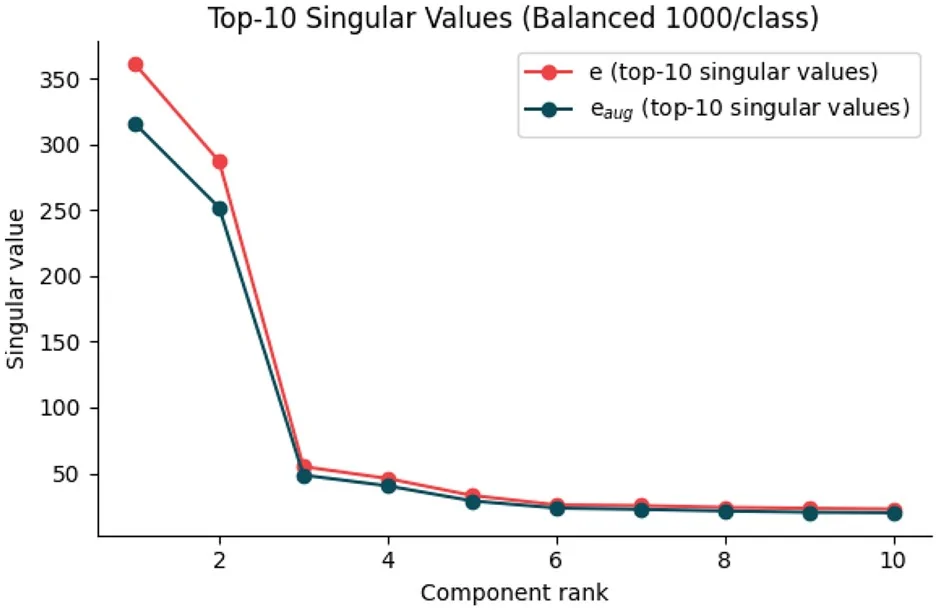
Singular value spectra of MiLaSol’s latent space reveal differences in effective dimensionality between *e* (before mixup) and $e_{\mathrm{aug}}$ (after mixup).

Prior work has shown that a lower singular value spectrum corresponds to a more information-dense representation (Verma *et al.* 2019). Compared to *e* (MiLaSol’s latent space before applying mixup), the augmented latent space $e_{\mathrm{aug}}$ exhibits smaller singular values and faster spectral decay (Fig. 2), indicating a more compact representation.

### 3.3 Model training

#### 3.3.1 Optimization framework

We train MiLaSol with AdamW (Kingma and Ba 2014) (lr = $1\times 10^{-4}$, weight decay = $1\times 10^{-5}$) coupled with a lookahead meta optimizer (Zhang *et al.* 2019). Learning rates follow a cosine annealing schedule with a warm restart every 8 epochs. Training terminates early if after the 8th epoch, no improvement was seen for three subsequent epochs.

#### 3.3.2 Loss function design

The training objective uses a composite loss that balances classification performance and representation quality by integrating supervised classification, contrastive learning, and reconstruction with entropy, prototype, and center regularization. Learnable weights adaptively balance these components during training, yielding a unified objective that promotes robust feature learning and improved generalization.


(2) $$\begin{array}{c} L_{\text{total}}=L_{\text{cls}}+\lambda_{\text{con}}L_{\text{con}}+\lambda_{\;\text{rec}}L_{\text{rec}}+L_{\text{regu}} \end{array}$$

where $L_{\mathrm{cls}}$, $L_{\mathrm{con}}$, $L_{\mathrm{rec}}$, and $L_{\mathrm{regu}}$ represent standard classification, contrastive, reconstruction, and regularization losses, respectively.

##### *3.3.2.1 Contrastive loss(* $L_{\mathrm{con}}$ *)*

Beyond traditional classification objectives, recent advancements in representation learning have highlighted the efficacy of contrastive approaches, such as Supervised Contrastive Learning (Khosla *et al.* 2020). This algorithm has demonstrated a noteworthy improvement in accuracy, achieving an enhancement of 8% on the ImageNet benchmark, which is widely regarded as the foremost evaluation standard in the field of computer vision. Such findings underscore the potential of contrastive learning to learn more discriminative representations. In our setting, the contrastive term operates over the solubility labels rather than protein family or domain identity: it pulls representations of soluble proteins toward one another and insoluble proteins toward one another, while pushing the two groups apart in representation space. To counter class imbalance, we weight the contrastive term by class frequency, analogously to the weighting available in nn. BCEWithLogitsLoss. We conduct an extensive sweep over the contrastive loss in this model.

##### *3.3.2.2 Regularization loss (* $L_{\text{regu}}$ *)*

We employ prototype-based learning (Snell *et al.* 2017) to enhance class-specific representation clustering through learnable class prototypes. Rather than one prototype per class, as is common in many-class settings, we keep a single prototype for the minority (negative) class. It is updated dynamically using exponential moving averages (proto=0.75×batch_mean+0.25×proto_prev) to stabilize evolution during training. Cosine embedding loss encourages negative class samples to approach their corresponding prototype, promoting intra-class compactness and more discriminative feature representations.

##### 3.3.2.3 Training and evaluation protocol

We trained our model on the complete DeepSol training dataset and evaluated performance on the standard test set, enabling direct comparison with existing methods. The benchmark enforces a 30% sequence similarity cutoff between train and test to limit data leakage. Our approach employs the same train-validation split as DeepSol. Final evaluation was conducted on the same independent benchmark test set used by DeepSol and PaRSnIP, ensuring fair comparison.

### 3.4 Hyperparameter tuning

Given the multi-component architecture of MiLaSol, we performed a hyperparameter search over a constrained grid to ensure stable training and fair comparison across configurations. Results appear in Tables S1 and S8, available at supplementary material  *Bioinformatics Advances* online. The batch size was fixed at 32 and the learning rate at $1\times 10^{-4}$. For the 1D CNN layers, kernel sizes of 5 and 7 were evaluated, with the number of convolutional filters varied between 128 and 192. The BiLSTM module used a single layer throughout, with hidden dimensions varying between 64, 128, and 192. The latent embedding dimension was fixed at 64. To balance auxiliary objectives with the primary binary classification loss, the supervised contrastive loss weight was swept from 0.1 to 0.9. We provide a detailed set of ablations in Figs S3 and S4, available at supplementary material  *Bioinformatics Advances* online.

## 4 Experimental setup

We test our methods and competitors on a standard benchmark dataset from Khurana *et al.* (2018). We utilize the same data splits as DeepSol, which comprises a total of 62 478 protein sequences for training, including 36 403 insoluble and 26 075 soluble sequences. The validation dataset consists of 6942 protein sequences, with 4045 classified as insoluble and 2897 as soluble. The test dataset contains 2001 protein sequences from Chang *et al.* (2014), with 1001 categorized as insoluble and 1000 as soluble.

We additionally compare against the recent PLM_Sol (Zhang *et al.* 2024), reproducing their experimental setup with the dataset released in their GitHub repository: 70 031 training, 4000 validation, and 4000 test sequences (78 031 total; 31 581 insoluble, 46 450 soluble). This dataset was curated from UESolDS (Zhang *et al.* 2024) by first removing insoluble sequences with high similarity to soluble ones (>75% identity, >70% coverage) to prevent leakage, then applying MMseqs2 (Hauser *et al.* 2016) clustering at 25% identity and 70% coverage to deduplicate the remainder.

### 4.1 Evaluation metrics

We follow the same evaluation criteria proposed by DeepSol (Khurana *et al.* 2018) focusing on accuracy, Matthew Correlation Coefficient (MCC), and the precision and recall of both classes. For our ablations (see the Supplement, available at supplementary material  *Bioinformatics Advances* online) we also report the AUROC and AUPRC’s derived from the precision recall scores.

## 5 Results

### 5.1 MiLaSol outperforms existing methods for protein solubility prediction

**Figure 3:**
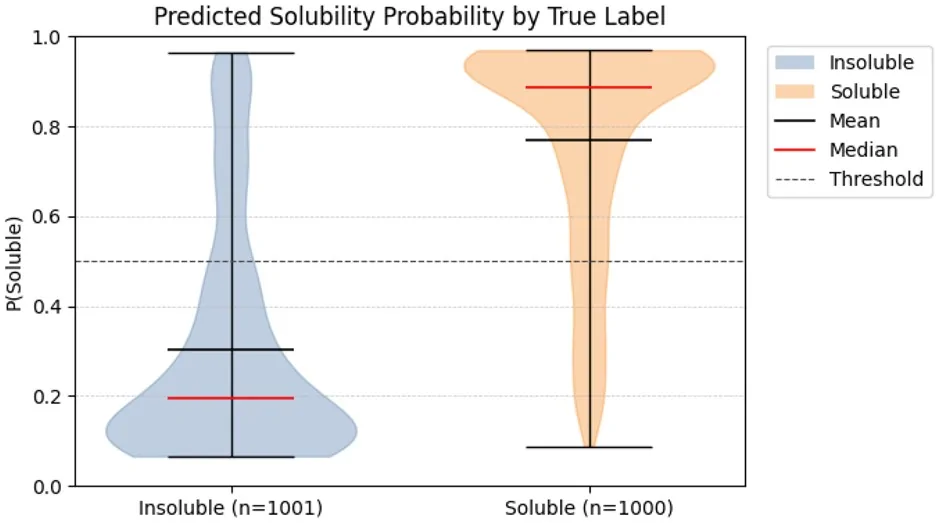
Predicted probability by true label. Horizontal lines show mean, median, and extrema; the dashed line marks the 0.5 decision threshold.

By integrating multiple PLM embeddings rather than relying on handcrafted features, MiLaSol achieves 81% accuracy on the standard DeepSol benchmark, exceeding all prior reported methods. Figure 3 illustrates the model’s discriminative behavior: insoluble proteins are consistently assigned predicted probabilities near 0.2, while soluble proteins cluster near 1.0, with clean separation around the 0.5 decision threshold.

On the standard DeepSol test set (Chang *et al.* 2014), MiLaSol attains the highest accuracy (0.81) and Matthews Correlation Coefficient (0.63) among all compared methods (Table 2). Selectivity and sensitivity are balanced across the soluble and insoluble classes, indicating that the improvement does not stem from a bias toward either label.

**Table 2:** Comparison of solubility prediction methods on key performance metrics on the benchmark dataset in Rawi et al. (2018).

Methods	Acc.	MCC	Sel. (insol.)	Sel. (sol.)	Sens. (insol.)	Sens. (sol.)	Gain (insol.)	Gain (sol.)
SCM	0.60	0.21	0.65	0.57	0.42	0.77	1.30	1.14
RPSP	0.52	0.03	0.52	0.51	0.44	0.59	1.03	1.02
PROSO	0.58	0.16	0.58	0.57	0.54	0.62	1.17	1.15
SOLpro	0.60	0.20	0.62	0.58	0.51	0.69	1.24	1.17
CCSOL	0.54	0.08	0.54	0.54	0.51	0.57	1.09	1.08
PROSO II	0.64	0.34	0.72	0.60	0.46	0.82	1.45	1.21
CamSol	0.52	0.07	0.59	0.51	0.13	0.91	0.26	**1.82**
PaRSnIP	0.74	0.48	0.75	0.74	0.73	0.75	1.50	1.47
DeepSol S1	0.73	0.46	0.75	0.71	0.69	0.77	1.50	1.42
PLM_Sol	0.76	0.52	0.80	0.73	0.70	0.82	1.39	1.65
DeepSol S2	0.77	0.55	**0.84**	0.72	0.65	**0.88**	**1.69**	1.44
DeepSol S3	0.77	0.54	0.81	0.73	0.69	0.84	1.63	1.46
SWI	0.51	0.04	0.54	0.51	0.19	0.84	0.39	1.67
EPSOL	0.79	0.58	0.79	0.79	0.79	0.79	1.58	1.57
MiLaSol	**0.81**	**0.63**	0.82	**0.81**	**0.80**	0.83	1.60	1.65

Best result in bold.

We therefore additionally evaluated MiLaSol on the more recent UESolDS-derived benchmark released with PLM_Sol (Zhang *et al.* 2024). As shown in Table 3, MiLaSol outperforms PLM_Sol on its own benchmark in four of seven metrics. The largest gaps are in precision (0.8107 vs. 0.6919) and specificity (0.8485 vs. 0.6308). When MiLaSol calls a protein soluble, it is less prone to misclassifying insoluble proteins as soluble. These gains carry through to higher overall accuracy (0.7488 vs. 0.7299) and a higher MCC (0.5077 vs. 0.4690), where MCC is perhaps the most informative single-number summary since it balances performance on both classes. PLM_Sol attains higher recall (0.8289 vs. 0.6490) and a marginally higher AUC (0.8342 vs. 0.8273), reflecting a more permissive operating point that recovers more true soluble proteins at the cost of many more false positives.

**Table 3:** Performance comparison on the second benchmark dataset, between PLM_Sol and MiLaSol.

Model	AUC	Acc	F1	MCC	Prec	Sens	Spec
PLM_Sol	**0.8342**	0.7299	**0.7542**	0.4690	0.6919	**0.8289**	0.6308
MiLaSol	0.8273	**0.7488**	0.7209	**0.5077**	**0.8107**	0.6490	**0.8485**

Performance tested on the UESolDS benchmark (Zhang *et al.* 2024) with balanced soluble and insoluble protein classes. Best values in bold.

### 5.2 Complementary PLM signals enhance solubility prediction

#### 5.2.1 Individual model contributions

Table 4 shows ablation study results comparing three protein language models: ESM2, ProtT5, and Raygun. Combining all three embeddings yielded the strongest performance across most metrics, indicating that each model captures complementary information collectively outperforming any individual approach. Figures S5 and S6, available at supplementary material  *Bioinformatics Advances* online in the supplementary material provide detailed visualizations of these results.

**Table 4:** Ablation of embedding inputs (ESM2, ProtT5, Raygun), with the sequence input held constant in all models on the main benchmark

Model Inputs	Acc.	MCC	Sel. (sol.)	Sel. (insol.)	Sens. (sol.)	Sens. (insol.)	Gain (sol.)	Gain (insol.)	AUROC	AUPRC
ProtT5	0.76	0.52	0.76	0.76	0.76	0.76	1.51	1.53	0.85	0.85
Raygun, ProtT5	0.77	0.54	0.77	0.76	0.76	0.78	1.52	1.55	0.86	0.85
Raygun	0.77	0.55	0.76	0.79	0.80	0.74	1.61	1.48	0.86	0.85
ESM2	0.78	0.56	0.79	0.77	0.77	0.80	1.53	1.59	0.86	0.86
ESM2, ProtT5	0.78	0.57	0.76	0.81	**0.82**	0.75	**1.64**	1.50	0.87	0.86
ESM2, Raygun	0.79	0.59	0.79	0.80	0.81	0.78	1.62	1.56	0.87	0.84
ESM2, Raygun, ProtT5	**0.81**	**0.63**	**0.82**	**0.81**	0.80	**0.83**	1.60	**1.65**	**0.89**	**0.87**

Best values in bold.

Next, we assessed individual model contributions by replacing each PLM with unit vectors. Figure S1, available at supplementary material  *Bioinformatics Advances* online reveals that ESM is consistently beneficial—removing it degraded performance according to all metrics, indicating its essential role. Figure S2, available at supplementary material  *Bioinformatics Advances* online quantifies these contributions by showing that ESM contributes the most overall, with the largest average performance loss observed when it was replaced with unit vectors. This finding underscores ESM’s critical importance compared to the other two PLMs.

#### 5.2.2 Denoising and regularization strategies

Beyond model selection, we examined the impact of training strategies by conducting ablation studies on the denoising autoencoder (DAE) and mixup approaches (Table 5). Label mixup degraded performance and was excluded from the final model. In contrast, latent mixup improved generalization without corrupting supervision and was retained.

**Table:** Ablation study on the main benchmark dataset evaluating the contributions of DAE, latent mixup, and label mixup.

Methods	Acc.	MCC
Full MiLaSol	**0.8151**	**0.6303**
MiLaSol (w/o DAE)	0.8141	0.6283
MiLaSol (w/o Latent Mixup)	0.7806	0.5625
MiLaSol (plus Label Mixup)	0.7931	0.5877

Compared with all ablated variants, the full MiLaSol model demonstrates the best overall performance. Best values in bold.

We further examined the contribution of the auxiliary regularization losses ($L_{\text{regu}}$) and of the latent space itself. Removing $L_{\text{regu}}$ entirely changes every metric by less than 0.01 (Table S9, available at supplementary material  *Bioinformatics Advances* online), so its quantitative contribution to accuracy is minor. To test how much of the task is already solved at the projection layer, we froze the backbone and replaced the MLP head with a single logistic-regression layer. On both the full model and the no-regularization model, aggregate metrics (Acc, MCC) stay within 0.02 of the full network, while the class-conditional gains diverge more (Table S10, available at supplementary material  *Bioinformatics Advances* online). The regularization losses therefore appear to shape the decision boundary,ΔGain(sol.)=0.138 with them versus 0.056 without, rather than drive overall accuracy.

### 5.3 Designing soluble protein variants with MiLaSol

We leverage MiLaSol’s rapid and accurate solubility predictor as an oracle to guide sequence design. A common approach to oracle-guided sequence optimization is simulated annealing (Karchin *et al.* 2005, Feyfant *et al.* 2007, Webb and Sali 2016). However, these methods quickly encounter a combinatorial explosion due to the vast space of possible insertion, deletion, and substitution operations, rendering them impractical for longer protein sequences.

To overcome this limitation, we exploit MiLaSol’s Raygun backbone, whose key advantage is a fixed-length latent representation of protein sequences (Devkota *et al.* 2024). Operating directly in this latent space enables efficient exploration of sequence neighborhoods without explicitly enumerating discrete mutation operations.

Our approach to designing soluble variants begins with proteins predicted to be insoluble. For each such sequence, we use Raygun to generate candidate variants by sampling from the latent space with Gaussian noise (standard deviation 0.1). To ensure evolutionary plausibility, we compute the latent-space distance between each generated sequence and the original input, retaining only candidates within a predefined threshold of 15. This threshold corresponds to approximately three times the expected L2 distance between two latent vectors with per-dimension standard deviation 0.1.

We then apply simulated annealing with five iterative optimization steps and 100 independent restarts to explore the latent neighborhood efficiently. At each step, candidate sequences are evaluated using MiLaSol, and optimization is guided by a temperature-dependent acceptance criterion. Finally, we retain sequences predicted to be soluble for downstream analysis, including structure prediction and experimental validation. Full algorithmic details are provided in Algorithm **S1** in the Supplementary Information.

### 5.4 Experimental motivation and dataset construction

Protein solubility is typically measured using cell lysates, where proteins detected in the soluble fraction are labeled as soluble (Smialowski *et al.* 2007). Ideally, a computational protein design method that aims to convert insoluble proteins into soluble ones would be evaluated on experimental datasets that explicitly measure solubility gains resulting from targeted mutations. However, to our knowledge, no such datasets currently exist.

Instead, available experimental data largely consist of deep mutational scans (DMS) (Araya and Fowler 2011), which typically assess the solubility effects of mutations relative to an already soluble wild-type protein. Our key insight is that we can approximate our desired evaluation setting *by considering DMS data in the reverse direction*: mutations that render an otherwise soluble protein undetectable in the soluble fraction. We use such data to construct an appropriate proxy database for evaluation of our method by collecting such examples from SoluProtMutDB (Veleckỳ *et al*. 2022), a curated database that aggregates multiple solubility-focused DMS experiments and provides a unified scoring system that enables cross-dataset comparisons. To construct an initial set of insoluble proteins, we select mutants with the most negative solubility scores labeled as *significantly deteriorating*. These sequences serve as starting points for MiLaSol-guided solubility optimization.

### 5.5 Sequence preparation

**Figure 4:**
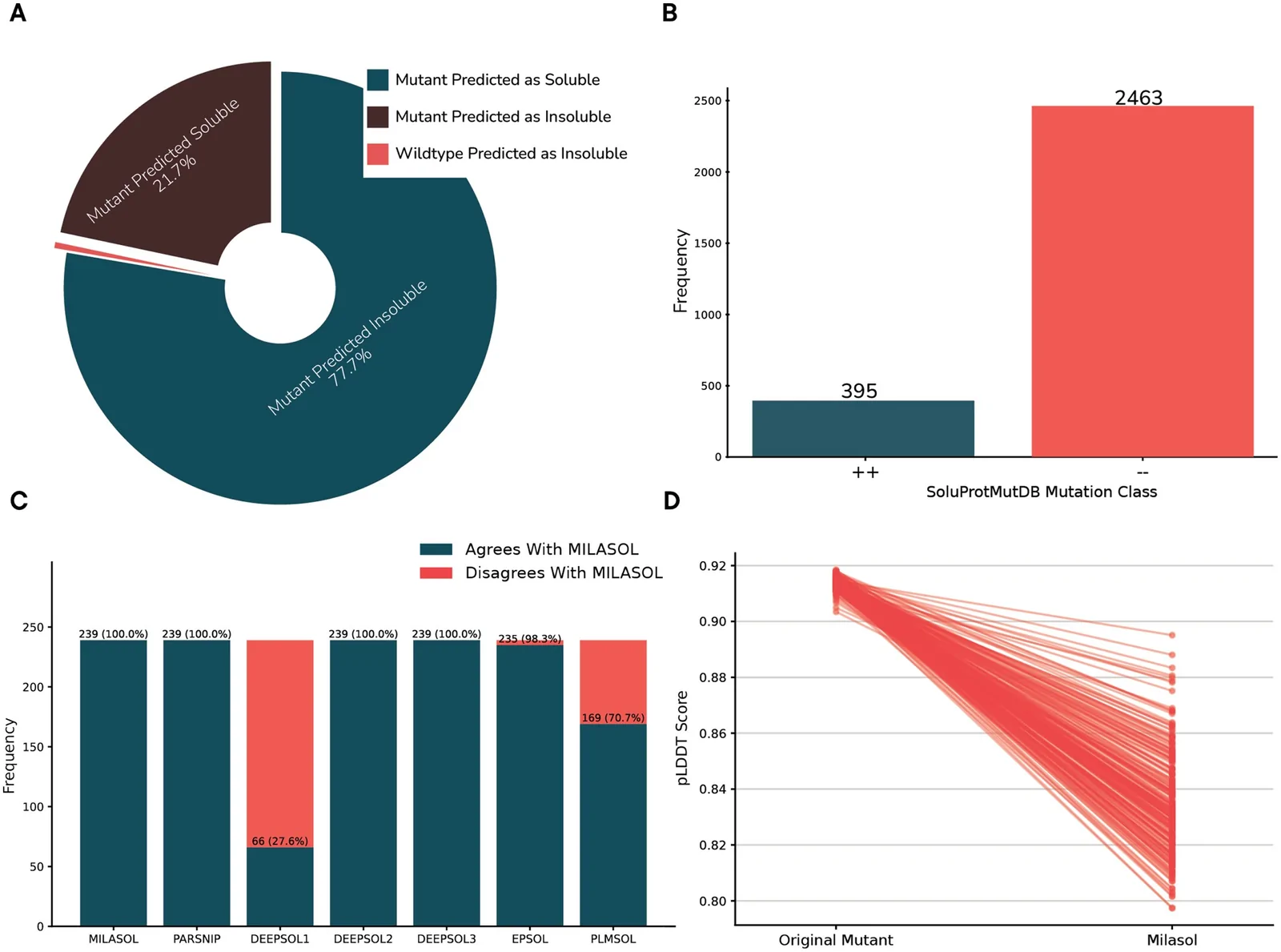
(A) MiLaSol predictions across SoluProtMutDB indicate that most mutant sequences are predicted to remain soluble, with only 21.7% classified as insoluble. (B) Among predicted insoluble mutants, the majority correspond to real world significantly deteriorating annotations (–). (C) PaRSnIP, DeepSol2/3, Epsol and PLM_Sol broadly agree with MiLaSol’s designed proteins. (D) Boltz-2 pLDDT scores demonstrate that solubility optimization preserves high-confidence folding, with all generated sequences remaining above pLDDT = 0.8.

We narrowed candidate sequences from SoluProtMutDB (Veleckỳ *et al*. 2022) by first applying MiLaSol to the entire database to obtain solubility predictions for both wild-type and mutant proteins. As shown in Fig. 4A, 77.7% mutant sequences were predicted to remain soluble, while 21.7% (2858 mutant sequences) were predicted to be insoluble.

We next examined the experimentally reported solubility effects of mutants predicted as insoluble (Fig. 4B). Of the 2858 mutants predicted to be insoluble, 2463 were annotated as significantly deteriorating, corresponding to mutations that reduce protein solubility by at least 33%. We choose the 2463 “– –” labeled sequences as our initial insoluble set.

### 5.6 MiLaSol guided simulated annealing produces plausible soluble sequences

We selected 1993 sequences from the 2463 predicted insoluble mutated proteins marked with “– –” (most decreasing solubility) annotated solubility effects. Using the MiLaSol predictor as a target function to maximize, we applied simulated annealing to enhance the solubility of these 1993 sequences. For each sequence, we performed five iterative optimization steps and repeated the process 100 times.

We iteratively sampled candidate sequences using Raygun’s noised sampler and evaluated proposed mutations with a temperature-dependent Metropolis criterion. We additionally enforced that the proposed sequences remain close to the original sequence by implementing a latent-space distance cap. The temperature was gradually annealed, to produce our final sequences.

Of the 1993 insoluble sequences, 239 were re-predicted as soluble by MiLaSol after simulated annealing—representing 12% of sequences rescued. Next, we compared MiLaSol’s predictions against PaRSnIP, DeepSol1/2/3, Epsol, PLM_Sol for the 239 reengineered proteins. Most methods demonstrated full/high agreement with MiLaSol (Fig. 4C)). We additionally validate our results by analyzing the plausibility of folding on these generated sequences. For each sequence we run Boltz-2 (Passaro *et al.* 2025) on default parameters and compare the predicted Local Distance Difference Test (pLDDT, see Supplementary Information, available at supplementary material  *Bioinformatics Advances* online) of the original mutant sequence and our generated sequences (Fig. 4D). We find that in all instances pLDDT drops slightly but remains above 0.8, except in one case as shown in the figure, maintaining a high score that indicates that the model is confident that the designed protein will still fold (according to thresholds as calibrated in Jumper *et al.* (2021)).

To characterize the redesign itself, we compared each insoluble starting sequence with its MiLaSol-designed soluble counterpart. On average 12.13% of positions were changed per pair, and the pairs remain conservative overall (mean BLOSUM62 raw score 1264.72; mean normalized score 0.8608). The edits are not uniform across residue types: polar residues were mutated most frequently (16.63% of their occurrences, versus 10.9%–11.4% for hydrophobic, acidic, and basic residues), and mutated positions were most often replaced by basic (37.1%) or polar (36.1%) residues, with acidic destinations rare (2.2%). Full per-category rates are given in Table S12, available at supplementary material  *Bioinformatics Advances* online in the Supplementary Information.

## 6 Discussion

We have introduced MiLaSol, a new deep learning method to predict solubility that by leveraging multiple protein language model embeddings can improve protein solubility prediction from previous methods. We next explored if MiLaSol could further leverage its embeddings to design soluble protein variants. Our results demonstrate the effectiveness of combining machine learning-based solubility prediction with generative optimization for protein engineering. The MiLaSol architecture’s ability to mix up multiple protein language models into an information-rich feature space may also improve other classification tasks that are dependent on protein structure.

## Supplementary Material

vbag233_Supplementary_Data

## Data Availability

MiLaSol is available at https://github.com/weiweiloutufts/milasol An archived version of the code at the time of submission can be found at https://doi.org/10.5281/zenodo.21495202
