# Supplementary material for: MiLaSol: modeling protein solubility by mixing up multiple protein language models
Source: Bioinform Adv. 2026 Aug 13;6(1):vbag233. doi: 10.1093/bioadv/vbag233 (PMC13581264; doi:10.1093/bioadv/vbag233)
Supplement: Supplementary file 1 [file vbag233_supplementary_data.pdf]

Supplementary Material for: MiLaSol: Modeling Protein Solubility by Mixing Up Multiple Protein Language Models

Weiwei Lou   Mert Erden   Lenore Cowen

Department of Computer Science
Tufts University
Medford, MA 02155

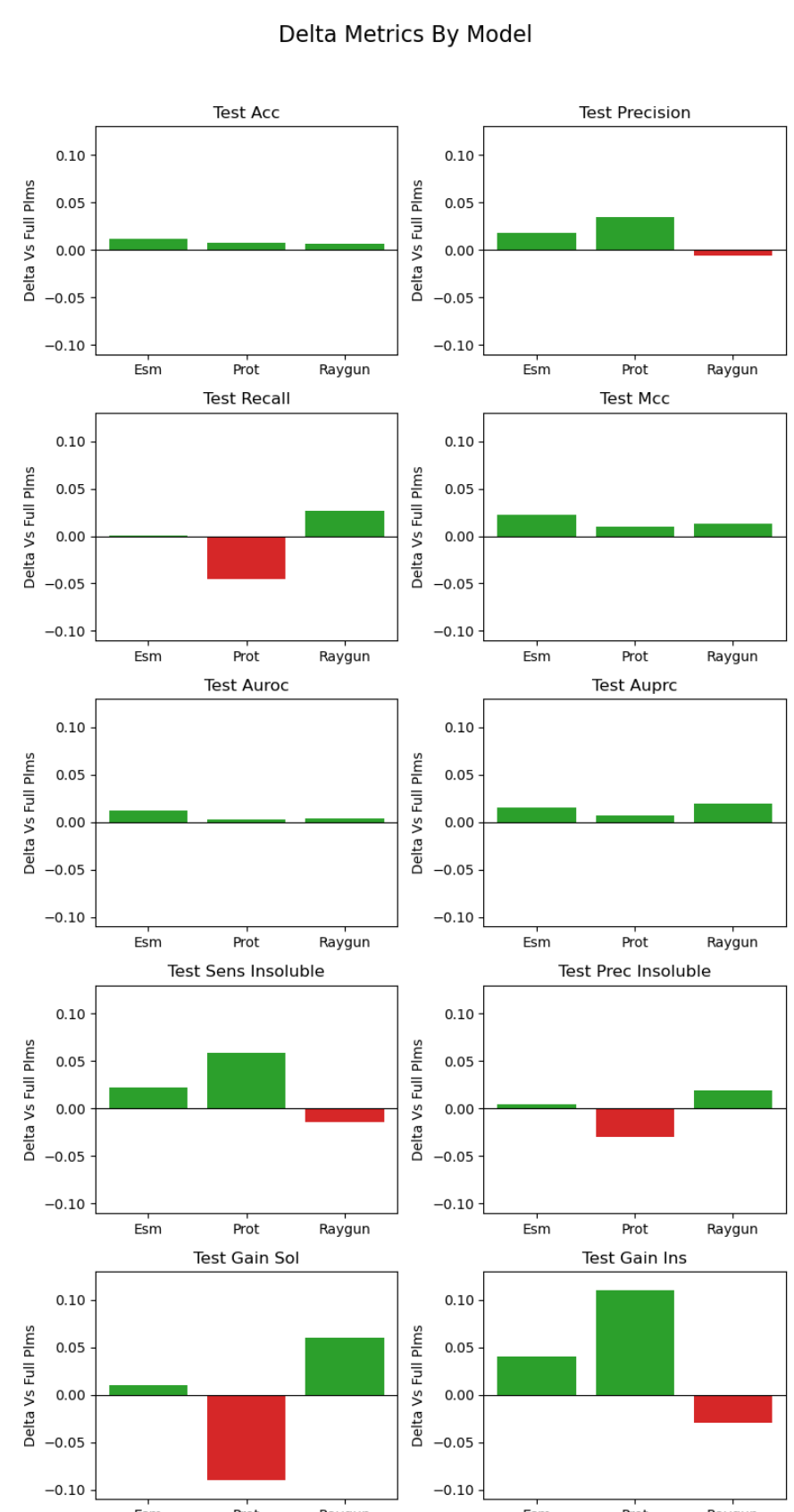

Figure S1: Per-Metric Impact of PLM Replacement. Green (above the X axis) indicates the full model (all 3 PLMs) performs best according to that metric; red (below the X axis) indicates that the ablation that replaces the named PLM with a unit vector is better than the full model on that metric. Including ESM is consistently beneficial across all metrics, while RayGun and ProtT5 show trade-off effects.

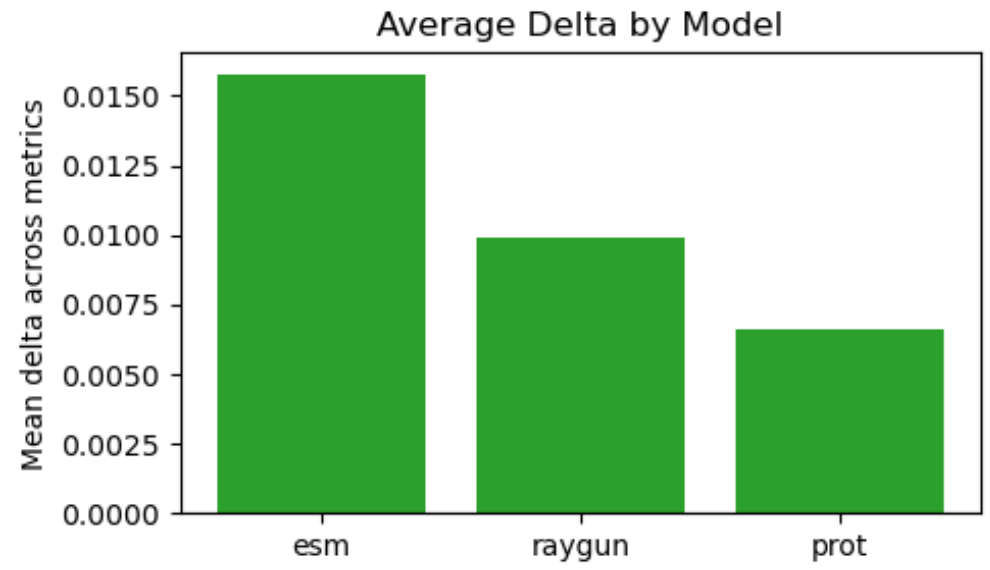

Figure S2: Average Performance Loss by PLM. ESM contributes most significantly to overall performance, with the largest average degradation upon replacement.

pLDDT Score

The pLDDT score is defined as the expected LDDT score, predicted by a neural network. It is a measure for how confident the model is of its own predictions. LDDT scores are defined as follows:

$$\text{LDDT}_i = \frac{1}{4N_i} \sum_{j \in \mathcal{N}(i)} \sum_{c \in \{\alpha_1, \alpha_2, \alpha_3, \alpha_4\}} \mathbf{1}\left(\left| d_{ij}^{\text{pred}} - d_{ij}^{\text{true}} \right| < c \right)$$

where $\mathcal{N}(i)$ are the neighboring residues of i within a 15 Angstrom cutoff, d_{ij} is the distance between residue i and j, and $\mathbf{1}$ is an indicator function that is 1 if the predicted distance and the ground true distance fall within a cutoff. Structure prediction tools are trained to predict these scores by replacing the indicator function with their own predicted probability:

$$\text{pLDDT}_i = \frac{1}{4N_i} \sum_{j \in \mathcal{N}(i)} \sum_{c \in \{\alpha_1, \alpha_2, \alpha_3, \alpha_4\}} P(|\Delta d_{ij}| < c)$$

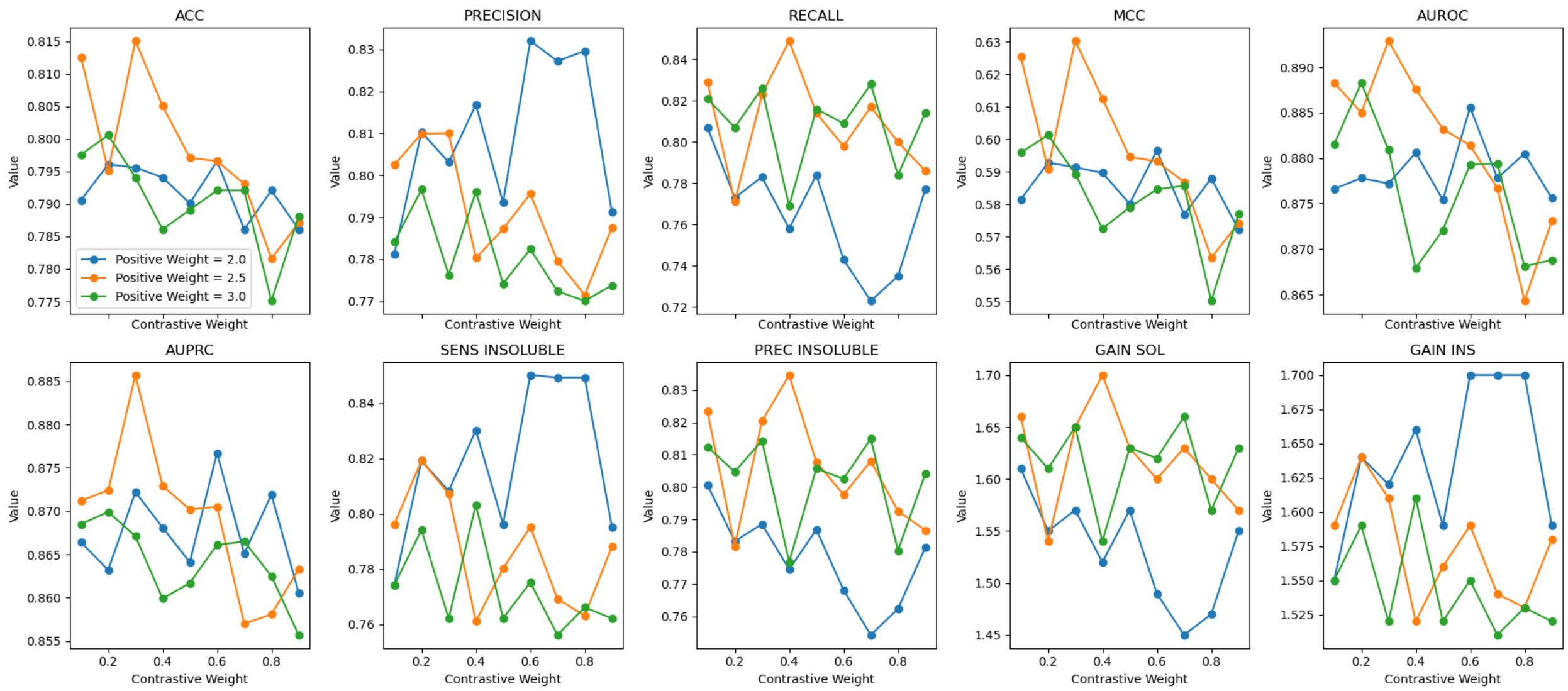

Figure S3: Hyperparameter sweep over contrastive-loss weight with three positive-class weighting settings (positive weight = 2.0, 2.5, 3.0). Curves show test-set performance across standard metrics (ACC, Precision, Recall, MCC, AUROC, AUPRC), insoluble-focused metrics (Sensitivity and Precision for insoluble proteins), and soluble/insoluble gain.

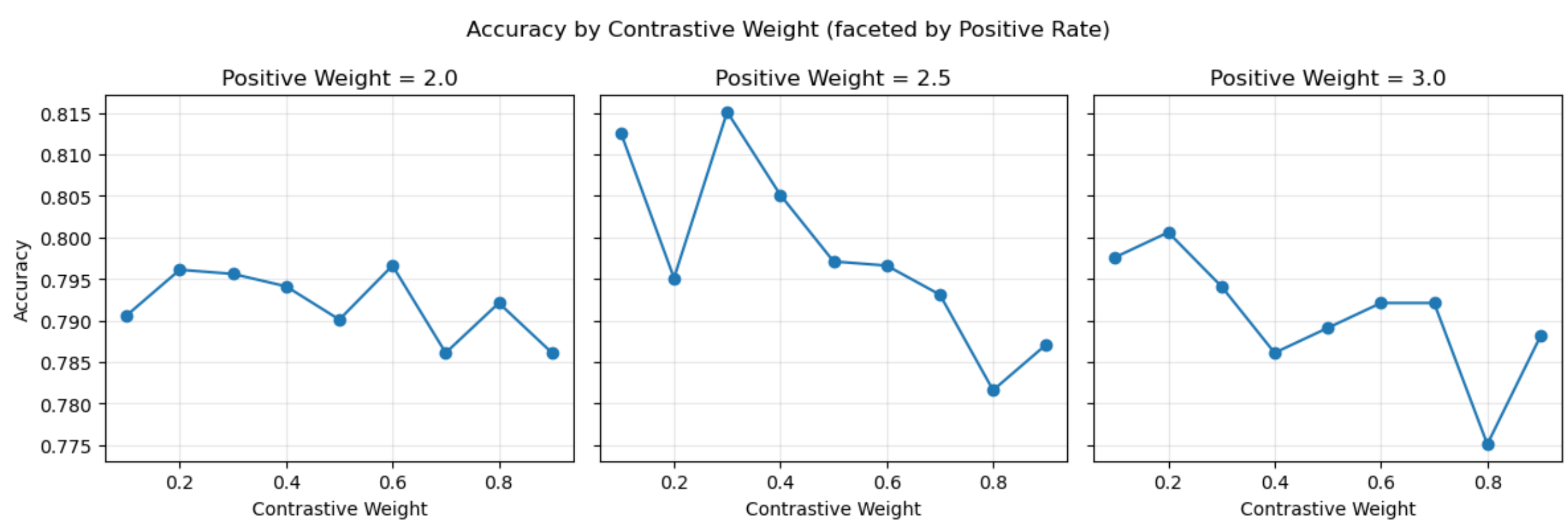

Figure S4: Zooming in on the test-set ACC performance for the hyperparameter sweep over contrastive-loss weight with three positive-class weighting settings (positive weight = 2.0, 2.5, 3.0) (top left panel of Figure S3).

Algorithm S1 PROTEIN_DESIGN: Simulated annealing with MiLaSol scores, Raygun proposals, latent-distance filtering, and Metropolis acceptance (single sequence)

Input: MiLaSol predictor ; initial sequence $s^{(0)}$; Raygun generator; steps n_{steps}; temperatures $\{T_t\}$; noise σ; restarts R; latent cap $D_{\max}$; clamp c
Output: best sequence $s^\star$ and best score b

Initialization
$x \leftarrow MiLaSol(s^{(0)})$ ▷ MiLaSol-predicted initial score
$s \leftarrow s^{(0)}$ ▷ current sequence
$a \leftarrow x$ ▷ current score
$s^\star \leftarrow s^{(0)}$; $b \leftarrow x$ ▷ best-so-far
$z^{(0)} \leftarrow Raygun(s^{(0)}; \sigma = 0)$ ▷ reference latent for distance cap

for $r \leftarrow 1$ **to** $\max(1, R)$ **do** ▷ optional restarts
  $s \leftarrow s^{(0)}$; $a \leftarrow x$ ▷ reset state
  for $t \leftarrow 1$ **to** n_{steps} **do** ▷ annealing schedule
    $T \leftarrow T_t$ ▷ current temperature

    Proposal(Raygun)
    $(s', z') \leftarrow Raygun(s; 0.1)$ ▷ propose a new sequence

    Latent-distance filter
    $d \leftarrow \|z^{(0)} - z'\|_2$ ▷ norm over flattened latent
    if $d > D_{\max}$ **then**
      $s' \leftarrow s$ ▷ reject: keep current sequence
      $z' \leftarrow z^{(0)}$ ▷ optional: keep within cap
    end if

    Scoring (MiLaSol)
    $y \leftarrow MiLaSol(s')$ ▷ proposed score
    $\Delta \leftarrow y - a$ ▷ score improvement

    Metropolis acceptance
    $p \leftarrow \exp\Big(\text{clip}(\Delta / \max(T, 10^{-12}), -c, c)\Big)$
    sample $u \sim \mathcal{U}(0, 1)$
    if $\Delta \geq 0$ **or** $u < p$ **then**
      $s \leftarrow s'$; $a \leftarrow y$ ▷ accept: update current state
      if $a > b$ **then**
        $b \leftarrow a$; $s^\star \leftarrow s$ ▷ update best-so-far
      end if
    end if
  end for
end for

return $s^\star, b$

Table S1: Kernel_size: 5, Num_filters: 128, CNN_embedding: 128.

Kernel	CNN Dim	Filters	LSTM Dim	Pos Rate	Loss	Acc	Prec	Recall	MCC	AUROC	AUPRC	Sens_{ins}	Prec_{ins}	Gain_{sol}	Gain_{ins}
5	128	128	64	2.0	0.4790	0.7741	0.8086	0.718	0.5517	0.8654	0.8479	0.8302	0.7466	1.44	1.66
5	128	128	64	2.5	0.4658	0.7881	0.7835	0.796	0.5763	0.8751	0.8610	0.7802	0.7929	1.59	1.56
5	128	128	64	3.0	0.4744	0.7746	0.7427	0.840	0.5540	0.8678	0.8513	0.7093	0.8161	1.68	1.42
5	128	128	128	2.0	0.4546	0.7901	0.8098	0.758	0.5814	0.8783	0.8691	0.8222	0.7728	1.52	1.64
**5**	**128**	**128**	**128**	**2.5**	**0.4359**	**0.8041**	**0.8187**	**0.781**	**0.6088**	**0.8857**	**0.8729**	**0.8272**	**0.7908**	**1.56**	**1.65**
5	128	128	128	3.0	0.4687	0.7886	0.7649	0.833	0.5795	0.8773	0.8686	0.7443	0.8169	1.67	1.49
5	128	128	192	2.0	0.4368	0.8021	0.8247	0.767	0.6057	0.8893	0.8777	0.8372	0.7824	1.53	1.67
5	128	128	192	2.5	0.4633	0.7886	0.7782	0.807	0.5776	0.8766	0.8719	0.7702	0.7998	1.61	1.54
5	128	128	192	3.0	0.4367	0.8006	0.7978	0.805	0.6012	0.8879	0.8796	0.7962	0.8034	1.61	1.59

Table S2: Kernel_size: 5, Num_filters: 128, CNN_embedding: 64.

Kernel	CNN Dim	Filters	LSTM Dim	Pos Rate	Loss	Acc	Prec	Recall	MCC	AUROC	AUPRC	Sens_{ins}	Prec_{ins}	Gain_{sol}	Gain_{ins}
5	64	128	64	2.0	0.4687	0.7851	0.7920	0.773	0.5704	0.8688	0.8596	0.7972	0.7785	1.55	1.59
5	64	128	64	2.5	0.5138	0.7601	0.7500	0.780	0.5207	0.8476	0.8261	0.7403	0.7711	1.56	1.48
5	64	128	64	3.0	0.4538	0.7896	0.7555	0.856	0.5844	0.8789	0.8625	0.7233	0.8341	1.71	1.45
**5**	**64**	**128**	**128**	**2.0**	**0.4409**	**0.8021**	**0.7909**	**0.821**	**0.6046**	**0.8817**	**0.8690**	**0.7832**	**0.8141**	**1.64**	**1.57**
5	64	128	128	2.5	0.4488	0.7946	0.8027	0.781	0.5894	0.8831	0.8731	0.8082	0.7870	1.56	1.62
5	64	128	128	3.0	0.4945	0.7836	0.7739	0.801	0.5676	0.8669	0.8626	0.7662	0.7940	1.60	1.53
5	64	128	192	2.0	0.4617	0.7856	0.8060	0.752	0.5725	0.8772	0.8698	0.8192	0.7678	1.50	1.64
5	64	128	192	2.5	0.4584	0.7866	0.8064	0.754	0.5744	0.8769	0.8639	0.8192	0.7692	1.51	1.64
5	64	128	192	3.0	0.4579	0.7931	0.7625	0.851	0.5902	0.8852	0.8784	0.7353	0.8316	1.70	1.47

Table S3: Kernel_size: 5, Num_filters: 192, CNN_embedding: 128.

Kernel	CNN Dim	Filters	LSTM Dim	Pos Rate	Loss	Acc	Prec	Recall	MCC	AUROC	AUPRC	Sens_{ins}	Prec_{ins}	Gain_{sol}	Gain_{ins}
5	128	192	64	2.0	0.4400	0.7916	0.7918	0.791	0.5832	0.8797	0.8613	0.7922	0.7914	1.58	1.58
5	128	192	64	2.5	0.4762	0.7751	0.7913	0.747	0.5511	0.8703	0.8624	0.8032	0.7606	1.49	1.61
5	128	192	64	3.0	0.4927	0.7811	0.7583	0.825	0.5644	0.8677	0.8557	0.7373	0.8083	1.65	1.47
5	128	192	128	2.0	0.4419	0.7886	0.8374	0.716	0.5833	0.8853	0.8762	0.8611	0.7522	1.43	1.72
5	128	192	128	2.5	0.4459	0.8016	0.8099	0.788	0.6034	0.8839	0.8737	0.8152	0.7938	1.58	1.63
5	128	192	128	3.0	0.4782	0.7881	0.7692	0.823	0.5776	0.8770	0.8675	0.7532	0.8099	1.65	1.51
5	128	192	192	2.0	0.4566	0.7816	0.8203	0.721	0.5674	0.8803	0.8713	0.8422	0.7513	1.44	1.68
**5**	**128**	**192**	**192**	**2.5**	**0.4355**	**0.8071**	**0.8034**	**0.813**	**0.6142**	**0.8918**	**0.8836**	**0.8012**	**0.8109**	**1.63**	**1.60**
5	128	192	192	3.0	0.5170	0.7681	0.7729	0.759	0.5363	0.8627	0.8539	0.7772	0.7635	1.52	1.55

Table S4: Kernel_size: 5, Num_filters: 192, CNN_embedding: 64.

Kernel	CNN Dim	Filters	LSTM Dim	Pos Rate	Loss	Acc	Prec	Recall	MCC	AUROC	AUPRC	Sens_{ins}	Prec_{ins}	Gain_{sol}	Gain_{ins}
5	64	192	64	2.0	0.4497	0.7911	0.7963	0.782	0.5823	0.8767	0.8642	0.8002	0.7861	1.56	1.60
**5**	**64**	**192**	**64**	**2.5**	**0.4443**	**0.8006**	**0.8069**	**0.790**	**0.6013**	**0.8832**	**0.8709**	**0.8112**	**0.7945**	**1.58**	**1.62**
5	64	192	64	3.0	0.5274	0.7631	0.7271	0.842	0.5330	0.8468	0.8151	0.6843	0.8126	1.68	1.37
5	64	192	128	2.0	0.4426	0.7921	0.8100	0.763	0.5852	0.8832	0.8746	0.8212	0.7762	1.53	1.64
5	64	192	128	2.5	0.4584	0.7856	0.8040	0.755	0.5723	0.8795	0.8694	0.8162	0.7693	1.51	1.63
5	64	192	128	3.0	0.4729	0.7906	0.7712	0.826	0.5827	0.8707	0.8491	0.7552	0.8129	1.65	1.51
5	64	192	192	2.0	0.4396	0.7956	0.8251	0.750	0.5936	0.8865	0.8779	0.8412	0.7711	1.50	1.68
5	64	192	192	2.5	0.4769	0.7781	0.7848	0.766	0.5564	0.8665	0.8607	0.7902	0.7717	1.53	1.58
5	64	192	192	3.0	0.4646	0.7861	0.7531	0.851	0.5771	0.8810	0.8746	0.7213	0.8289	1.70	1.44

Table S5: Kernel_size: 7, Num_filters: 128, CNN_embedding: 128.

Kernel	CNN Dim	Filters	LSTM Dim	Pos Rate	Loss	Acc	Prec	Recall	MCC	AUROC	AUPRC	Sens_{ins}	Prec_{ins}	Gain_{sol}	Gain_{ins}
7	128	128	64	2.0	0.4774	0.7836	0.8189	0.728	0.5707	0.8723	0.8571	0.8392	0.7554	1.46	1.68
7	128	128	64	2.5	0.4614	0.7921	0.8120	0.760	0.5854	0.8719	0.8450	0.8242	0.7746	1.52	1.65
7	128	128	64	3.0	0.4723	0.7886	0.7929	0.781	0.5773	0.8707	0.8569	0.7962	0.7844	1.56	1.59
7	128	128	128	2.0	0.4676	0.7691	0.8064	0.708	0.5422	0.8692	0.8573	0.8302	0.7400	1.42	1.66
7	128	128	128	2.5	0.4609	0.7971	0.8049	0.784	0.5944	0.8792	0.8747	0.8102	0.7897	1.57	1.62
7	128	128	128	3.0	0.4765	0.7831	0.7625	0.822	0.5680	0.8657	0.8489	0.7443	0.8072	1.64	1.49
**7**	**128**	**128**	**192**	**2.0**	**0.4332**	**0.8086**	**0.8164**	**0.796**	**0.6174**	**0.8869**	**0.8733**	**0.8212**	**0.8012**	**1.59**	**1.64**
7	128	128	192	2.5	0.4693	0.7901	0.7810	0.806	0.5805	0.8731	0.8647	0.7742	0.7998	1.61	1.55
7	128	128	192	3.0	0.4529	0.7991	0.7848	0.824	0.5990	0.8798	0.8719	0.7742	0.8149	1.65	1.55

Table S6: Kernel_size: 7, Num_filters: 128, CNN_embedding: 64.

Kernel	CNN Dim	Filters	LSTM Dim	Pos Rate	Loss	Acc	Prec	Recall	MCC	AUROC	AUPRC	Sens_{ins}	Prec_{ins}	Gain_{sol}	Gain_{ins}
7	64	128	64	2.0	0.4538	0.7861	0.7918	0.776	0.5723	0.8743	0.8627	0.7962	0.7806	1.55	1.59
7	64	128	64	2.5	0.4510	0.7926	0.7865	0.803	0.5853	0.8787	0.8618	0.7822	0.7990	1.61	1.56
7	64	128	64	3.0	0.5159	0.7616	0.7460	0.793	0.5243	0.8493	0.8298	0.7303	0.7793	1.59	1.46
7	64	128	128	2.0	0.4765	0.7681	0.7797	0.747	0.5367	0.8653	0.8524	0.7892	0.7574	1.49	1.58
7	64	128	128	2.5	0.4539	0.7781	0.8366	0.691	0.5648	0.8789	0.8684	0.8651	0.7370	1.38	1.73
7	64	128	128	3.0	0.4672	0.7821	0.8032	0.747	0.5656	0.8761	0.8655	0.8172	0.7638	1.49	1.63
7	64	128	192	2.0	0.4593	0.7786	0.7749	0.785	0.5573	0.8751	0.8666	0.7722	0.7824	1.57	1.54
7	64	128	192	2.5	0.4809	0.7726	0.7512	0.815	0.5472	0.8625	0.8508	0.7303	0.7980	1.63	1.46
**7**	**64**	**128**	**192**	**3.0**	**0.4239**	**0.8131**	**0.8027**	**0.830**	**0.6266**	**0.8939**	**0.8802**	**0.7962**	**0.8242**	**1.66**	**1.59**

Table S7: Kernel_size: 7, Num_filters: 192, CNN_embedding: 128.

Kernel	CNN Dim	Filters	LSTM Dim	Pos Rate	Loss	Acc	Prec	Recall	MCC	AUROC	AUPRC	Sens_{ins}	Prec_{ins}	Gain_{sol}	Gain_{ins}
7	128	192	64	2.0	0.4515	0.7916	0.8008	0.776	0.5835	0.8752	0.8607	0.8072	0.7829	1.55	1.61
7	128	192	64	2.5	0.4843	0.7856	0.7922	0.774	0.5714	0.8620	0.8430	0.7972	0.7793	1.55	1.59
7	128	192	64	3.0	0.4786	0.7841	0.7863	0.780	0.5682	0.8663	0.8593	0.7882	0.7820	1.56	1.58
7	128	192	128	2.0	0.4605	0.7831	0.8137	0.734	0.5689	0.8726	0.8619	0.8322	0.7580	1.47	1.66
7	128	192	128	2.5	0.5132	0.7681	0.7939	0.724	0.5383	0.8564	0.8448	0.8122	0.7466	1.45	1.62
**7**	**128**	**192**	**128**	**3.0**	**0.4378**	**0.7981**	**0.7849**	**0.821**	**0.5968**	**0.8877**	**0.8769**	**0.7752**	**0.8126**	**1.64**	**1.55**
7	128	192	192	2.0	0.4448	0.7881	0.8186	0.740	0.5789	0.8840	0.8793	0.8362	0.7630	1.48	1.67
7	128	192	192	2.5	0.4831	0.7781	0.7914	0.755	0.5568	0.8653	0.8489	0.8012	0.7660	1.51	1.60
7	128	192	192	3.0	0.4733	0.7736	0.7805	0.761	0.5474	0.8678	0.8665	0.7862	0.7671	1.52	1.57

Table S8: Kernel_size: 7, Num_filters: 192, CNN_embedding: 64.

Kernel	CNN Dim	Filters	LSTM Dim	Pos Rate	Loss	Acc	Prec	Recall	MCC	AUROC	AUPRC	Sens_{ins}	Prec_{ins}	Gain_{sol}	Gain_{ins}
7	64	192	64	2.0	0.4811	0.7756	0.8120	0.717	0.5550	0.8689	0.8590	0.8342	0.7469	1.43	1.67
7	64	192	64	2.5	0.4835	0.7786	0.8057	0.734	0.5594	0.8655	0.8507	0.8232	0.7560	1.47	1.65
7	64	192	64	3.0	0.4883	0.7801	0.7708	0.797	0.5606	0.8696	0.8578	0.7632	0.7901	1.59	1.53
7	64	192	128	2.0	0.5126	0.7611	0.7862	0.717	0.5242	0.8568	0.8434	0.8052	0.7401	1.43	1.61
7	64	192	128	2.5	0.4701	0.7841	0.7591	0.832	0.5709	0.8695	0.8500	0.7363	0.8144	1.66	1.47
**7**	**64**	**192**	**128**	**3.0**	**0.4568**	**0.7976**	**0.7752**	**0.838**	**0.5972**	**0.8847**	**0.8729**	**0.7572**	**0.8239**	**1.68**	**1.51**
7	64	192	192	2.0	0.4480	0.7891	0.8176	0.744	0.5805	0.8795	0.8678	0.8342	0.7654	1.49	1.67
7	64	192	192	2.5	0.4687	0.7951	0.7831	0.816	0.5907	0.8738	0.8635	0.7742	0.8081	1.63	1.55
7	64	192	192	3.0	0.4753	0.7891	0.7642	0.836	0.5808	0.8729	0.8595	0.7423	0.8192	1.67	1.48

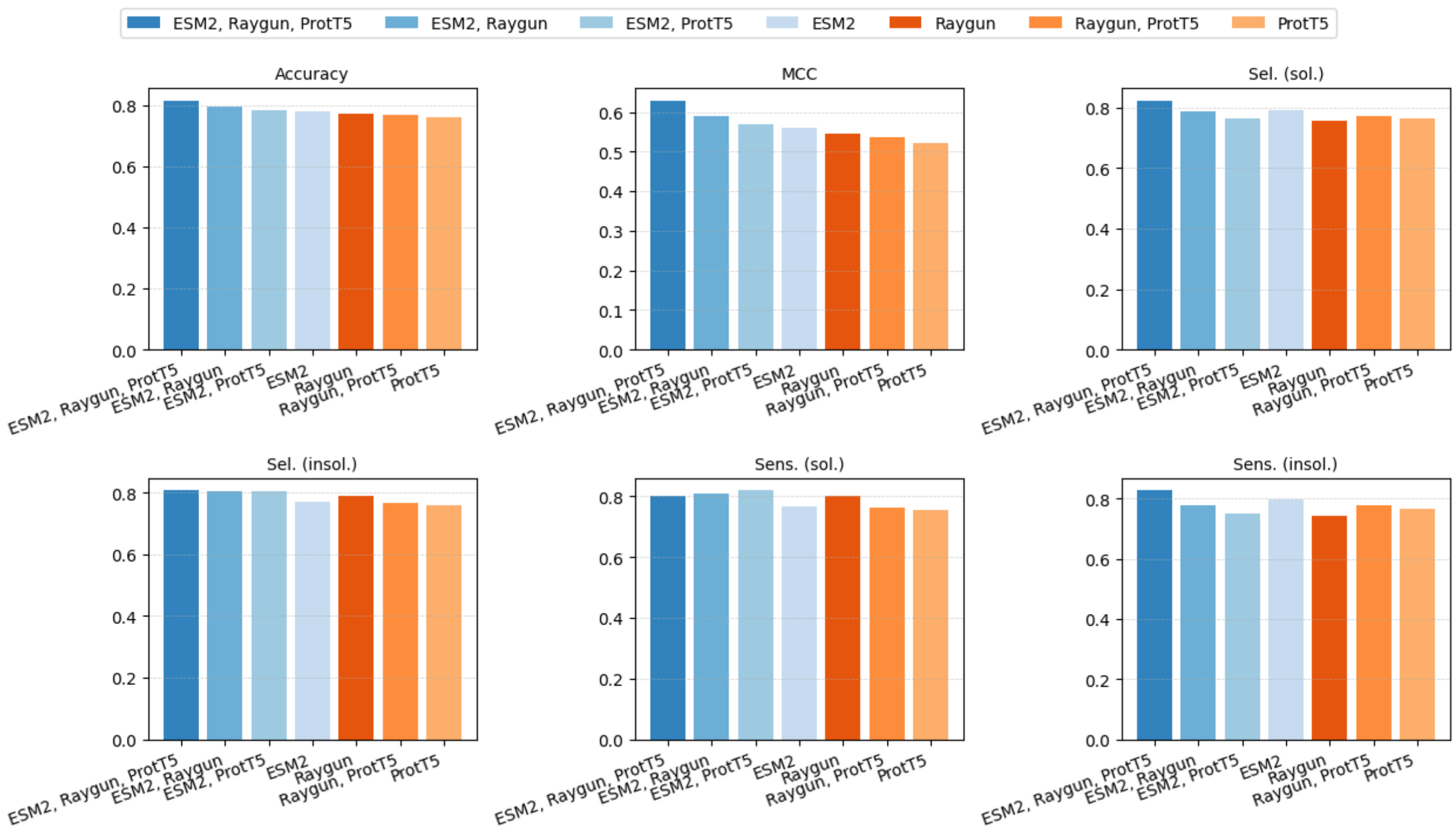

Figure S5: MiLaSol with different input embeddings (ESM2, ProtT5, RayGun). Bars show test metrics: Accuracy,Selectivity (Soluble),Selectivity (Insoluble),Mcc,Sensitivity (Soluble),Sensitivity (Insoluble).

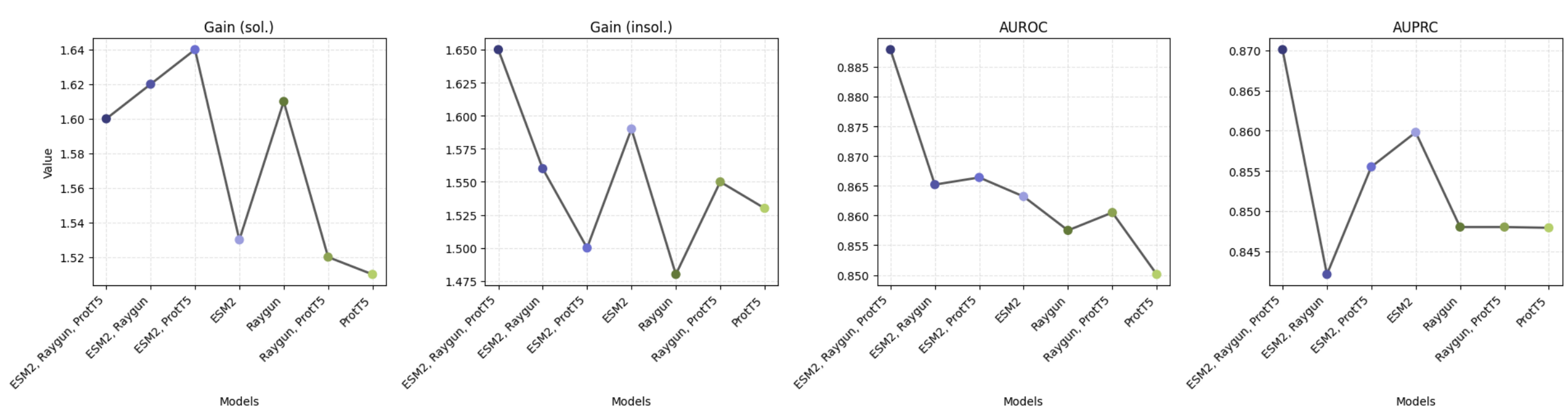

Figure S6: MiLaSol with different input embeddings (ESM2, ProtT5, RayGun). Points show test metrics: Soluble Gain,Insoluble Gain,AUROC,AUPRC.

Loss-Component and Latent-Space Ablations

Removing the auxiliary regularization losses ($\mathcal{L}_{\text{regu}}$) changes every metric on the main benchmark by less than 0.01 (Table S9), indicating a minor quantitative contribution to overall performance. To probe how much of the task is already solved at the projection layer, we froze the backbone and replaced the MLP head with a single logistic-regression layer. For both the full model and the no-regularization model, aggregate metrics (Acc, MCC) stay within 0.02 of the full network, while the class-conditional gains diverge more (Table S10). The divergence is larger with the regularization losses in place (ΔGain(sol.) $= 0.138$) than without them (ΔGain(sol.) $= 0.056$), suggesting these losses shape the decision boundary rather than drive overall accuracy.

Table S9: Effect of removing the regularization losses ($\mathcal{L}_{\text{regu}}$) on the main benchmark. All metrics change within 0.01.

Variant	Acc	MCC	Sel. (sol.)	Sel. (insol.)	Sens. (sol.)	Sens. (insol.)	Gain (sol.)	Gain (insol.)
Full model	0.8151	0.6303	0.8203	0.8100	0.8072	0.8230	1.6100	1.6500
No $\mathcal{L}_{\text{regu}}$	0.8126	0.6253	0.8181	0.8073	0.8042	0.8210	1.6076	1.6428
Δ	0.0025	0.0050	0.0022	0.0027	0.0030	0.0020	0.0024	0.0072

Table S10: Linear-probe ablation. With the backbone frozen, the two-layer MLP head is replaced by a single logistic-regression layer, for both the full model and the model trained without the regularization losses. Aggregate metrics (Acc, MCC) stay within 0.02 while the class-conditional gains diverge more.

Variant	Acc	MCC	Sel. (sol.)	Sel. (insol.)	Sens. (sol.)	Sens. (insol.)	Gain (sol.)	Gain (insol.)
Full model	0.8151	0.6303	0.8203	0.8100	0.8072	0.8230	1.6100	1.6500
+ linear probe	0.8036	0.6128	0.8510	0.7674	0.7363	0.8710	1.4718	1.7429
Δ	0.0115	0.0175	−0.0307	0.0426	0.0709	−0.0480	0.1382	−0.0929
No-regu model	0.8126	0.6253	0.8181	0.8073	0.8042	0.8210	1.6076	1.6428
+ linear probe	0.8096	0.6206	0.8319	0.7901	0.7762	0.8430	1.5517	1.6868
Δ	0.0030	0.0047	−0.0138	0.0172	0.0280	−0.0220	0.0559	−0.0440

Contribution of the Tokenized Sequence Input

The three PLM embeddings used by MiLaSol are pooled, sequence-level representations that capture global context but discard residue-level positional detail. The tokenized amino-acid sequence is retained as a separate input so that the CNN and BiLSTM can recover this local context. Table S11 quantifies the effect

of removing the tokenized sequence entirely, leaving only the three pooled PLM embeddings: accuracy drops from 0.81 to 0.70 and MCC from 0.63 to 0.42.

Table S11: Ablation removing the tokenized amino-acid sequence input, leaving only the three pooled PLM embeddings (ESM2, Raygun, ProtT5). Full-model values are reproduced from the main results.

Variant	Acc	Prec	Recall	F1	MCC	AUROC	AUPRC	Sens. (insol.)	Prec. (insol.)	Gain (sol.)	Gain (insol.)
MiLaSol (full)	0.81	0.82	0.80	0.81	0.63	0.89	0.87	0.83	0.81	1.60	1.65
w/o tokenized AA seq	0.70	0.67	0.79	0.73	0.42	0.78	0.75	0.61	0.75	1.59	1.23

Redesigned-Residue Analysis

Across the 239 MiLaSol-designed soluble variants and their insoluble starting sequences, on average 12.13% of positions were changed per pair, and the pairs remain conservative overall (mean BLOSUM62 raw score 1264.72; mean normalized score 0.8608). Table S12 reports the mutation rate by original residue category and the destination category of mutated residues. Polar residues were mutated most frequently, and mutated positions were most often replaced by basic or polar residues, with acidic destinations rare.

Table S12: Redesigned-residue statistics across the 239 MiLaSol-designed soluble variants. (a) Mutation rate by original residue category (mutated / total occurrences). (b) Destination category of mutated residues.

(a) Mutation rate by original residue category

Category	Mutated / Total	Rate
Hydrophobic	4082 / 37293	10.95%
Polar	2267 / 13632	16.63%
Acidic	981 / 8590	11.42%
Basic	960 / 8839	10.86%

(b) Destination category of mutated residues

Category	Count	Share
Basic	3079	37.1%
Polar	2995	36.1%
Hydrophobic	1993	24.0%
Acidic	183	2.2%
Other	40	0.5%
